# Concept of Health and Sickness of the Spanish Gypsy Population: A Qualitative Approach

**DOI:** 10.3390/ijerph16224492

**Published:** 2019-11-14

**Authors:** Antonio Jesús Ramos-Morcillo, César Leal-Costa, César Hueso-Montoro, Rafael del-Pino-Casado, María Ruzafa-Martínez

**Affiliations:** 1 Department of Nursing, Faculty of Nursing, University of Murcia, 30100 Espinardo, Spain; cleal@um.es (C.L.-C.); maruzafa@um.es (M.R.-M.); 2 Faculty of Health Sciences, University of Granada, 18016 Granada, Spain; cesarhueso@ugr.es; 3 Department of Nursing, School of Health Sciences, University of Jaén, 23071 Jaén, Spain; rdelpino@ujaen.es

**Keywords:** Roma health, prevention, promotion health, healthcare cultural sensitivity, Roma values, ethnic groups, social class, healthcare disparities, Roma, Spain

## Abstract

The Roma community (RC) has poor health indicators, and providing them with adequate healthcare requires understanding their culture and cultural differences. Our objective was to understand the concept of the health and sickness of the RC in Spain, and for this, a qualitative study was conducted. A content analysis utilizing an inductive approach was used to analyze the data. Twenty-three semi-structured interviews were performed, and four main categories were obtained after the analysis of the data: perception of the state of health, the value of health, what was observed, and causal attribution. The inter-relations between the categories shows that the RC have a dichotomous worldview split between non-sickness (health) and sickness mediated by causal attribution. Their worldview is polarized into two values: not sick/sick. When not sick, optimism is prioritized along with happiness, and these two emotions are highly valued, as they also play a physical and social function. When a person becomes noticeably sick, this is understood as being in a negative and severe state, and when there are visible physical implications, then the need to act is made clear. When faced with the need to act, the behavior of the RC is mediated by causal attributions, influenced by nature and religion, timing, concealment by not mentioning the disease, and the origin of the healthcare information. For the organization of an adequate health response for the RC, it is necessary for healthcare systems to be able to merge culture and health care.

## 1. Introduction

The Roma community (RC) is the most numerous ethnic minority in Europe, with a population that oscillates between 10–12 million [1]; in Spain, there is a population numbering between 700,000 to 970,000 individuals [2]. The RC is found in a state of socio-economic disadvantage that translates into worse health conditions [3]. In Spain, this community tends to have a greater infant mortality [4], a worse health status [5], and a life expectancy that is 7 years below the mean of the general population [6,7]. However, until recently, the state of health of the RC has not been a specific subject of analysis [5,8,9]. The studies conducted have been mainly descriptive and quantitative, and focused on describing the most common health problems that affect this population, aspects related to their lifestyle, and their demand for health services [8]. An increase in the number of studies that propose interventions from the social context point of view [8], which include representatives of minority communities [10] or that improve the cultural competencies of the health professionals [11], have been observed, with a positive impact on some health indicators of the minorities being examined.

The relative advances in healthcare research until the present day have shown that inequalities exist, but these have not been sufficiently explained. Also, explanations have not been provided that justify why some benefits result from some interventions and not others, and it has been revealed that the RC has a need for health assistance for which a model of prevention that fits them does not exist [5]. Improving the health of this population requires research on the cultural differences of the RC related to their concept of health and sickness, utilizing qualitative methods [12]. The lack of knowledge about these aspects is considered a barrier for their adequate use by healthcare institutions and for their appropriate care by health professionals [13]. Also, in the explanatory models of health and sickness, cultural differences have been found to be among the communication problems with the health professionals [14]. Lastly, as pointed out by those who work with the Roma population, more training is needed on the meaning of sickness for this minority [15]. Delving into the experiences, beliefs and attitudes about health and sickness of the RC could explain their observed healthcare behavior, lifestyle and the demands placed on health services [16,17]. Ultimately, this knowledge could enable the development of health interventions that are specifically adapted to them, which could improve their behaviors related to the adherence to treatments, as well as recommendations about healthcare and healthcare systems [18,19]. Therefore, the objective of the present research study was to understand the concept of health and sickness of the RC in Spain, using a qualitative approach.

## 2. Materials and Methods 

### 2.1. Design 

A qualitative study was conducted, as it is valid for understanding the personal experiences of the participants, and it allows us to obtain a deeper understanding of the perceptions, beliefs and values of specific groups [20]. 

### 2.2. Participants

The research was conducted in the province of Jaén (Spain), in the cities of Úbeda, Linares and Jaén. Twenty-three adult individuals from the RC participated in the research study. A maximum variation sampling was utilized to establish the profiles of the sample, to guarantee a criteria of heterogeneity, and to grant the study greater sample richness. Thus, the following were taken into account: life course (without children, school-aged children, and with emancipated children), sex (men/women), level of education (no education, primary education, secondary education and university), work history (paid or not paid work) and evangelical church attendance.

The number of informants was defined by utilizing the criteria of saturation. This is a standardized method for estimating the size of a sample [21].

### 2.3. Data Collection

The collection of information took place from November 2013 to January 2016, through semi-structured interviews. The participants were interviewed in person by the members of the research team (AJR-M, MR-M). The research team had experience with qualitative research and were proficient with semi-structured interviews. A script with questions was first agreed upon by experts and posteriorly reviewed by RC leaders. This script consisted of two parts: the first part regarded socio-demographic characteristics, and the second part was related to the concept of health and sickness. The questions were formulated from the general to the specific. Situations related to health were posed, and subjects were asked to provide an opinion about them. For example, one of the questions was as follows: “What should a person who has high blood pressure or high sugar do?”.

The RC leaders and the social services facilitated the recruitment of the people interviewed. The participants were asked to specify the place, day and hour for their interview, with these being conducted in public places not related to the healthcare system (all of them in Social Centers, except for three performed in a Health Center due to an explicit request). No one refused to be interviewed, although on some occasions, some individuals missed their appointment. When this was the case, the person was replaced with another with the same characteristics. Field notes were taken after each interview. 

Thematic saturation was achieved as no new themes were identified in the data collection and the phenomenon was sufficiently explained [22]. 

### 2.4. Ethical Considerations

The project was approved by the research ethics committee of Jaén (Department of Health, 23022012). Each informant participated in the study voluntarily, after receiving information about the research, as well as the ethical and confidentiality guarantees. They could ask questions and provide observations about the study before the interview, and their consent was obtained afterwards. To ensure confidentiality, codes were used and the identification information was eliminated.

### 2.5. Data Analysis

Content analysis with an inductive approach was used to analyze the data [23]. The transcribed interviews were imported into NVIVO 8 (QSR International, Victoria, Australia) for the management and analysis of the data. Three main phases were executed: preparation, organization and presentation of reports.

In the preparation part, the interviews were selected as the unit of analysis, and the data were analyzed in depth to provide them with meaning, to understand “what is occurring” and to obtain meaning as a set. In the organization phase, an open codification was conducted, creating categories and abstractions. The transcriptions, categorizations and emergent themes were discussed by the research team (AJR-M, MR-M, CL-C) for their verification.

### 2.6. Validity and Reliability/Rigor

The reliability of the data was verified by comparing the codification by the three researchers who codified the transcripts (AJR-M, MR-M, CL-C) independently. Afterwards, the consistencies and discrepancies among the three researchers were determined. The disagreements were resolved by consensus.

All the researchers were aware of their personal opinions and biases. For this, they designed a document prior to the analysis in which they recorded these opinions and biases in writing. This document was always present physically in the analysis by each team member. The research team maintained a constant reflection throughout the process of analysis. The quotes that best represented the categories were extracted in order to present them in the results section.

The COREQ criteria were used as a guideline for reporting this qualitative study [24].

## 3. Results

Twenty-three interviews were performed with a mean length of 55 min. The characteristics of the informants can be seen in Table 1.

Four main categories were obtained after the analysis of the data: perception of the state of health, the value of health, what is observed, and causal attribution. Specific categories also have various sub-categories (Table 2). A detailed description of the categories, subcategories and vertbatims can be found in Appendix A.

### 3.1. Perception of the State of Health

The people interviewed had a remarkable optimism when they evaluated their state of health. Also, in many occasions, God was present in relation with health.

“I’m extremely well” JH1.

“Good, thank God” UH5.

Even when they had chronic diseases, with important comorbidities, they defined themselves as being in good health. At the same time, they used words that downplayed their negative health issues, such as “just”, “only” or “a little”.

“My health is good, it’s just the sugar” UH4.

In general, it could be said that a state of being sick appeared within a state of being healthy.

“If you are sick […], maybe you are healthy but something doesn’t work, it doesn’t mean that your health is not good because you have some sickness” LM2.

This optimism is an important pillar in their worldview of life, and it is vital for them. This idea is present in their entire discourse.

### 3.2. The Value of Health

Being healthy is highly valued. Frequently, health is shown to have a high social desirability, and it is more valued than possessing other social or material aspects.

“Well, I know that everyone will value health equally, but I know that in the gypsy community, one of their… I don’t know, of their foundations is health and freedom, which in the gypsy language, it is “sastipen tali” LM1.

Health is not only something that is desired for the individual, but for the family as well, and it is observed that having “yours” gathered and near is highly valued. This is how situations in which family and happiness are found together are understood.

“Your house, live happy with yours, for me, this is health” LM1.

### 3.3. What is Observed

Sickness has a marked negative character. Being sick implies a personal limitation that is associated with a great personal sadness. It is understood that being sick brings with it a lack of functionality. Even defining someone as sick could be offensive.

“[…] Because […] says; he’s sick, pff. I don’t like it, I don’t like that word, for them to say he’s sick, that’s what it is, I don’t like saying it. However unwell someone is, you don’t tell him he’s sick. In my point of view, (saying) one is unwell, but sick… For me it is a harsh word” UM3.

A very negative aspect of sickness is its physical consequences. The physical appearance that shows sickness is a reason for disgust, and this disgust has an important social content, as the ramifications of being sick are observed by the others. This limits the functionality and therefore the loss of social function.

“Being healthy is having good eyesight, being strong and having a good face color (appearance)” UM1.

The discourse of those interviewed is very polarized, where being sick signifies being severely ill or critical (even when this is not the case). This polarization is a mechanism that allows them to understand the world in a simple and dichotomous manner; either I am healthy or I am sick.

“The gypsy has suffered few illnesses, but as soon as he becomes unwell, he dies” JH1.

Health is the opposite of sickness. It is not possible to find a definition of health in the discourses. Health is defined from the concept of sickness or being sick. Although the term health has a positive connotation, those interviewed talk about it using negative terms. Health is understood as the opposite of sickness: I am healthy because I am not sick.

(Interviewer) How is your health?

(Interviewee) Good, but apparently, it hurts once in a while, because it seems that it is hereditary […]. As for the rest, I don’t have any complaints about anything. UH9.

Regarding the tangible and the need to act, when something is visible and therefore occurring, specific actions need to be performed. This implies that chronic illnesses may not be understood as such, and one could think that nothing is occurring when one does not have or show symptoms.

“I believe that when they get a shot (of insulin) and they are ok, they will say I’m fine now, I don’t need a shot, that’s what I think… they let themselves go a bit, and when they are unwell again, they get a shot again, and so on” UH2.

### 3.4. Causal Attribution

Generally, the individuals explain the events related to health and sickness to themselves and to others with vague ideas, with few specifics and without much depth.

Regarding nature being inexplicable and hazardous and its connection with God (and the consequences), the origins of disease and health are born from nature and from God. This origin is generally attributed to an inexplicable causality, which in many cases depends on chance.

“Life is like, it is a thing that is nature ” […] “I don’t understand that” […] ”Yes, yes, not that, not that, it’s just whatever has to happen, happens” UM3.

“What are they going to do? As if God was not the only one…” UH9.

The understanding of health and sickness as being linked to nature and God results in passivity in preventive actions against sickness.

“(To be healthy) I don’t do anything. It stays like this and that’s it” UM1.

Sickness and health are not something that could be understood through reflection, and this is not thought about much by the RC.

“And another thing, because God wants it. It’s just that… I don’t know what else to say.” LM1.

Regarding the idea that if something is not mentioned, it does not exist; this is an inverse causal attribution, and thus, if something is not mentioned with words, it must therefore not exist.

The fear of sickness is associated with an abstract misfortune whose identity is unknown, but frightens people a great deal. To speak about sickness (implying that they are severely sick) entails that they are close to death, and therefore, the word is avoided.

“I don’t know many who get x-rays or analysis, because for us, the less we know, the better, about the illnesses” […] “I don’t know, and I don’t want to know” UH2.

#### 3.4.1. Temporality

Temporality is present in a fundamental manner in the RC’s understanding of the concept of sickness and health. This temporality leads to focusing our attention on two moments in time, towards the now and towards the past, but not paying attention to the future.

This category has different facets: on the one hand, the value of the temporal moment itself (worry vs. acting), and on the other hand, the observation (temporal sequence) of what is occurring.

#### 3.4.2. Worry vs. Acting

This aspect is understood as “living in the present”, an intrinsic value of the RC. The focus of the healthcare actions is done in the “now”. It is when there is a problem “now” that I have to pay attention and do something about it.

“I see them worry a lot (the non-gypsies) due to many circumstances of life. What happens? When a person worries, he is preparing his mind for something bad to happen. It is completely the opposite of being an optimist. It is pessimistic. Worrying is a synonym of pessimism, and this creates stress in many of them […] The gypsies, well, we have a greater optimism. If something happens, we solve it, and that’s it. We take care of the circumstance, but we never think that something bad is going to happen to us” […] “The non-gypsy has too many worries, and does not act” JH1.

#### 3.4.3. Temporal Sequence

The world that surrounds us is observed. Keeping the timeline in mind, it is observed how the person is able to establish associations between events that occurred before and healthcare situations that occurred after and that associated to the previous event. Thus, for example, a relationship between being pregnant as a previous step before losing dentures is observed.

“With the belly, they fell” […] “part of the mouth becomes destroyed” UM1.

#### 3.4.4. Origin of the Information

It seems that a source of information about why things happen is “word of mouth”. Nevertheless, the Roma are not sure if things happen as explained by others.

“They say that a glass of wine daily is good, I’ve heard this from people” UH2.

Another important aspect is the acquisition of information through the religious pastor.

“The thing is, I asked the pastor of my church and he detected it, he says that I don’t have depression, it’s a bit of anxiety; doubts and fears” LM1.

Nevertheless, there is a polarized discourse with the figure of the pastor. A feeling is transmitted that he things depend on God, and therefore one does not have control, and therefore one should not do anything special to be healthy.

“The current drive for the gypsies to massively turn to religion and all is leading the gypsies to abandon themselves to nature” UH4.

**Figure 1:**
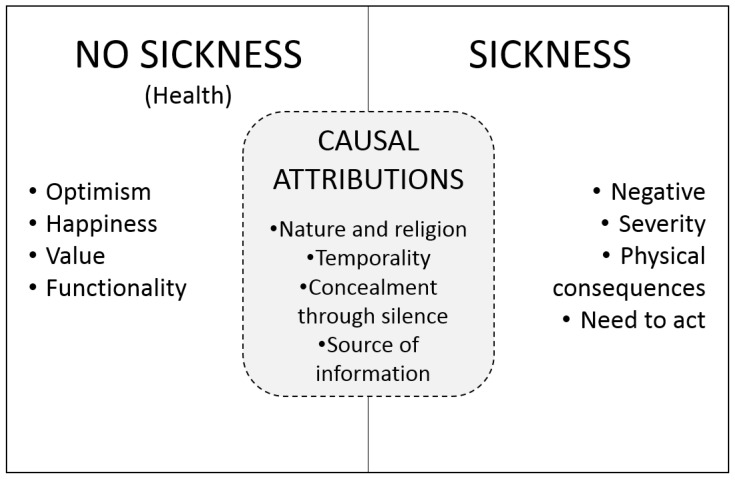
Worldview of the concept health/sickness of the Roma community.

The inter-relation between the categories found shows that the RC have a dichotomous worldview regarding non-sickness (health) and sickness mediated by causal attribution (Figure 1). Their worldview is polarized into two values: not sick/sick. When not sick, optimism is prioritized along with happiness, and these are highly valued, as they also possess a physical and social function. When it is observed that one is sick, it is understood that it is a severe, negative state of health, with visible physical ramifications, and this is when the need to act becomes clear. When faced with the need to act, the behavior of the RC is mediated by causal attributions, influenced by nature and religion, temporality, concealment by not mentioning the disease, and the origin of the information.

## 4. Discussion

The findings of this research describe the beliefs and meanings that the RC utilizes to make sense of their experience with health and sickness. The Spanish RC has its own view of health and sickness, which re-enforces the idea that there is no universal agreement about what “health” and “sickness” mean [25]. The RC does not define the concept of health, as pointed out by other studies [26], and health experiences are more intangible than the experiences of sickness. For the RC, health is a state that is identified with happiness and liberty, and it is valued by the individual as well as the family. The concept of health and sickness transcends the individual and interacts with the extended family. This could result in the individual’s decisions being conditioned by the extended family, resulting in a more complex relationship with healthcare systems. Studies indicate that this could have a direct effect on the clinical practice, as what the doctor discusses or agrees with the patient could be posteriorly altered by the extended family [27]. On the other hand, sickness is what “is seen”—a negative aspect of their personal and social life—and this is generally translated into a state of denial up to the limit. When the sickness is very severe and there are consequences, it is thus transformed into something tangible and visible, and one must therefore act. The causal attribution of the disease by the RC shows an external control locus (forces of nature, God), and it is also defined by what they are experiencing in the present. Thus, they avoid speaking about sickness or diseases. The health behavior theory shows us that these aspects determine their lifestyles, their perception of risk, and the moment in which they seek help, and thus their overall health behavior [28].

In light of these findings, the interpretation of some indicators of health of the RC should be revised. The perception of the state of health has been referred to by the RC as extremely good, as influenced by their optimistic view of life. Diverse studies have demonstrated that people who self-evaluate their state of health as very good or good utilize health services less, accumulate fewer health problems and have a lower probability of dying and becoming sick [29,30]. Paradoxically, international studies [6,31] show that the self-rated health of the RC in diverse European countries, even when they are practically identical to the European average, have much worse indicators of morbidity and mortality. This finding compels us to be cautious when making direct comparisons of the self-rated health of the RC with other groups, as this could be influenced by optimism and therefore be overestimated. Likewise, it may not be an adequate indicator with respect to the use of health services, as the value of this indicator in the general population and the RC is not associated with the same demand for a general practitioner [1]. Some nuances can be made from these findings. As shown by a comparative study between the general Spanish population and the Roma [5], the Roma population with a lower level of education, who are older than 35 and retired individuals, who are businesswomen and those who tend to the home have a worse self-perception of health. Also, especially in women, the deterioration of their self-perceived state of health due to age is faster and more severe in the Roma population.

The finding of the concept of sickness being opposite to health has been defined by other authors [32,33] and allows us to explain some health behaviors of the RC. Also, it shows us that this community understands their surroundings in a simplistic manner, and the explanation of health is basic: one either “has it or doesn’t have it”. This could be due to the lack of academic training, as 8.6% are illiterate (as compared to 2.2% of the total population in Spain), and 50.7% to 67.2% do not have a formal education [8,14]. On the other hand, it justifies behaviors observed in the RC, such that providing care is only for people who are sick, never for those who are healthy, and therefore health care should be immediate and decisive. This implies that if the visible part of the sickness has already disappeared, the disease has disappeared, and the therapy can be abandoned [32]. Also, this concept of sickness can be linked to the fact that they ask for medical assistance specifically when one is sick (or severe), which indicates, among other things, that they are not aware and are not able to utilize the portfolio of health services offered or do not know about the other health professional categories [16]. Also, this behavior would explain why the RC has a low level of medical consultations for aspects related to prevention [5].

Religion, as in other cultures, has an influence on the concept and managing of sickness of the RC [34]. The causal attribution of sickness is interpreted as a mixture between destiny, inexplicable chance, and the hand of God. However, although they mentioned that the disease could be due to God, it is always in a respectful manner, and they never referred to Him in a negative manner or placed blame. It is estimated that around 80% of the gypsy population knows and frequents the Evangelical Church [35], so that it is believed that this concept is very common in this community. Nevertheless, some studies have shown that the Evangelical Church can also act as a protection factor of the RC when teaching behaviors associated to the care of one’s health [27]. As shown in other studies [9], the external attribution of the causes of sickness favors the idea that the RC is not active in the search for and the maintenance of their health and that they delegate it to the figure of the doctor, resulting in a dependence on the healthcare system, with a great passivity towards the modification of lifestyles. Also, on some occasions, this behavior can be driven by and perpetuated in the healthcare systems by the type of relationship with the doctor, as patients from a lower social class receive more medical anamnesis, a more direct style of consultation, and a decreased level of shared making of decisions [36].

A key finding for the design of strategies for primary care is the importance of the timeline for the RC. In general, the RC takes care of the problems that are currently occurring, not of the problems that could occur later on. They do not think about the future, with respect to life or health. The translation of this finding is extremely relevant in the clinical context, as it conditions their use of healthcare services. Prevention-related consultations will be scarce [5,6] and health promotion programs tend not to work, as they are not understood and therefore do not make sense. Additionally, if the disease appears without any reason, then specific health behaviors are a waste of time. There are no arguments to introduce healthy practices that do not offer visible results. This finding could help justify why the main cause described at the European level for the lack of vaccination (41.9%) is “due to forgetfulness”; in Spain, the use of emergency services in the last 12 months is 37.4% [6], and the emergency services are utilized twice as much by the RC as social class I—the professionals with higher education [1]. 

Our findings are especially important with respect to their possible use in the development of health policies and programs, as well as the improvement of the cultural competencies of health professionals with respect to ethnic minorities such as the RC. Thus, different organizations should develop programs for their professional workers to sensitize and train them in cultural competencies [11], with particular stress on programs aimed at doctors and where the Roma population are part of the design [15]. Strategies should be designed that allow the RC to use all the services offered by the health services; currently, they only focus on those offered by their doctor [16]. Lastly, the health services should commit to being the drivers of efforts for achieving equality in the local context [16]. The health systems should promote primary care playing a leading role, as it is known that this is where the greatest degree of health of the population and fewer inequalities are achieved [37].

With respect to professional health workers, they should possess cultural competencies [11], avoiding medical care that performs an anamnesis that is too biomedical or a management-style consultation [36]. The professionals should take advantage of the contact with the RC to evaluate their health needs, in a detailed manner, taking into consideration a possible over-estimation of their state of health. The professionals tend to avoid the inverse care law [38] and become aware about it in their clinical activity. Likewise, it would be desirable that professionals should become able to manage the social inequalities in health within their consultation area [16].

Future research should be directed towards understanding the results that are obtained when the health policies and professionals take into account these explanatory models of health when designing health care services. At the same time, research is needed that is able to provide information about the perception of health and sickness related with (1) the influence of the sociodemographic characteristics retarding this perception and (2) the aspects of this concept which are due to the influence of the sociodemographic variables and which are due to the specific cultural characteristics of the RC. 

The limitations of the study are related, in the first place, to the design of the research, which does not allow extrapolation due to the lack of external validity. On the other hand, the access to the sample was very complicated. Although this access was managed through the associative network and social services, on many occasions, the informants did not attend the appointment. This could imply that the discourses analyzed could be partially different to those from the individuals who did not want to participate. Another limitation is the absence of literature regarding the concept of the health and sickness of the Spanish and international RC, which has not allowed for the complete discussion of the results.

## 5. Conclusions

The RC possesses a concept of health that is different from the “health” that health services usually cater to. This research facilitates the understanding of the notion of health, sickness and the causal attribution of sickness of the RC. This helps us to better understand many of the health-related behaviors observed and why prevention and health promotion services have a difficult path to travel with this ethnic group. For the organization of an adequate health response for the RC, it is necessary for health systems to be able to merge culture and health care. Also, we must be cautious in the interpretation and use of the health indicators utilized in the general population.

## Figures and Tables

**Table 1:** Characteristics of the informants and duration of the interviews.

*N*	Women	Age	Children	Work	Education	Evangelical Church	Duration
1	UM1	43	5	No	4th year BE *	Yes	56 min
2	UM2	22	1	No	1st year BE	No	48 min
3	UM3	43	4	Yes	2nd year BE	Yes	59 min
4	UM4	43	3	No	6th year BE	-	1 h 05 min
5	LM1	41	3	Yes	Vocational training	Yes	55 min
6	LM2	30	1	Yes	Bachelor	No	38 min
7	UM5	35	3	Occasional	3rd year BE	Yes	49 min
8	UM6	67	3	Retired	Illiterate	Yes /Little	35 min
9	UM7	69	2	Retired	Reading	Yes	40 min
10	JM1	47	4	Occasional	5th year BE	Yes	59 min
11	LM3	56	5	No	2nd year BE	Yes	1 h 15 min
	**Men**	**Age**	**Children**	**Work**	**Education**	**Evangelical Church**	**Duration**
1	UH1	47	4	Yes	4th year BE	Yes	60 min
2	UH2	18	0	Student	4th year BE	Yes	59 min
3	UH3	75	4	Retired	Reads and writes	No	47 min
4	UH4	57	5	Occasional	BE	No	1 h 03 min
5	JH1	39	3	Yes	Vocational training	Yes	1 h 19 min
6	UH5	74	5	Retired	Reads and writes	No	50 min
7	UH6	52	3	Occasional	5th year BE	Little	56 min
8	UH7	24	0	None	3rd SE **	No	39 min
9	UH8	35	1	Pensioner	4th SE	No	1 h 03 min
10	JH2	46	2	Occasional	5th year BE	Yes	58 min
11	LH1	59	4	Pensioner	2nd year BE	Yes	1 h 02 min
12	UH9	52	3	Occasional	5th year BE	Yes	55 min

* BE, basic education: primary school (6–14 years old), before 1995. ** SE, secondary education (12–16 years old), after 1995.

**Table 2:** Categories and subcategories.

Category	Subcategory
Perception of the state of health	---
The value of health	---
What is observed	Negative Physical consequences The severity Health: the opposite of sickness The tangible and the need to act
Causal attribution	Nature, inexplicable, hazardous and its connection with God (and the consequences) What is not mentioned does not exist The temporality: - Worrying vs. acting - Temporal sequence Source of information

## Appendix A

**Table A1:** Categories: subcategories and verbatims.

Categories	Subcategories	Verbatims
Perception of the state of health	---	- Optimism for health: “I am excellently well” JH1 “I’ve never been bad” UM2 “well, thank God. What is sugar, sugar, right now, sugar and a little cholesterol” UH5 - Self-perception: “health, I’m fine, it’s light stuff, it’s blood pressure, women’s problems” UM5 “I health very well, just sugar” UH4 “(cholesterol) was a little high” UM9 - Disease within health “if you have a disease […], maybe you are healthy but something is wrong, it doesn’t mean that maybe because you have a disease it’s because your health is going very badly” LM2 - Vital optimism: “The gypsy himself is usually a happy person, because the more ignorant you are, the happier you are. That’s true, that’s how it is. In their ignorance the gypsies are happy because they are satisfied with little, they live a day” […] “but that the gypsies in themselves are usually happy people, animated people” UH8
The value of health	---	- High social desirability: “man, I know that everyone will value health equally, equally, but I know that the gypsy community one of its… I who know, its foundations is health and freedom, which in Romani is often called “sastipen tali”“ LM1 “Most important of all is health” UM4 “So you’re in a good position, a good house, if you’re bad, what good is it to you? Not at all. UM4 “I health. Nothing more. That’s it. More than that word, they don’t take any more words, there are too many words” […] “health, more than money. […] because if you have millions and you are sick, why do you want money? Would you like to have a lot of money, a lot of it and be sick? What do you want better money or health? […] that’s it, there’s no more” UH5 “(health) Well, it’s everything in life. It is the maximum. Because a person who is healthy can walk around the world and look for his life and enter, leave, move, see your children to your grandchildren. I think that’s the greatest thing that exists today, more than money, more than anything!” UH9 - Health for the family: “Right now I feel like I’m fine, those in my house are fine right now” UM1 “your home, live happily, live with yours, for me that’s health” LM1 “Because a pot of whatever it is […] is made in the family, it is not made alone, because you cannot grill it, it impregnates feelings of loneliness in you, and even […] you feel bad, but when there is a paella and you share it, the smile comes to you as soon as you see it…” JH1
What is observed	● Negative	“Where am I going to go? What am I going to do? Concerns that, that… that cause you that discomfort” UH1 “For they enter into a deep sadness, let’s say, if they have no inducement… they suffer it, they live it within themselves…” UH1 “[…] because he says; he’s sick, poof. I don’t like it, this word I don’t like, they say it’s sick, that’s what I don’t like to say. No matter how bad it is and everything, you don’t say it to a person. To me it seems, (to say) that it is bad, that… but sick… For me it is a strong word.” UM3 “That person is going to go into depression. That’s what I wouldn’t want him to say to his face (the doctor he’s sick to). Or they are… there are a lot of doctors who are very abrupt” UH9
	● Physical consequences	“you get very dry”, “you swell with the medications you take, with everything “UM2 “Not being able to do what you want or what you need. In other words, unable to do what you must” UH1 “to be healthy is to have good eyesight, to be strong, to have good face color” UM1
	● The severity	“When a person is ill, he carries a high blood pressure […] he is quite serious. Call the doctor that there is a person who is ill, and who cannot wait” UH10 “The gypsy has suffered very few illnesses but it was getting sick and dying” JH1 “I have never been bad or anything I have not had anything operated on, I have had normal deliveries. UM1 “(Being in the hospital) and I heard him say: no, no, but you tell me, what is it, but is it good or bad? The doctor was talking to you, you’ve got this! And they summed it up but it’s good or bad. They sum up in that no, nothing happens to you that this is not going to die, then it’s not bad, is it? […] It’s all in a word that defines how healthy they are, you know?” UH8
	● Health, the opposite of sickness	(Interviewer) How is your health? (Interviewee) Good, apparently, it does hurt from time to time, because you can see that it is hereditary […]. I don’t complain about anything else. UH9
	● The tangible and the need to act	“And when it comes like that, diseases like these (measles outbreak), you know it’s going to get infected because we get the vaccine, we get vaccinated” UM5 “I believe that when they prick themselves (to put insulin) and they are well, they will say they are already well, I do not need to prick me, I say… They are already left a little and when they are bad again, they prick again and so on” UH2 “I think a doctor is there for you when something happens to send you medicine and try to cure you” UH9 “They know that if they eat other things, for example; salt, which harms him, or if they are diabetic things of, of, so sweet and that, can also harm them… but:: don’t think about it! Or if they think about it later with the medicine they see that nothing happens” UH9 “when there’s a close case is when, let’s say, that’s the information that directly reaches the soul because they know… what happens! They don’t see it as something far away that you tell them “UH8
Causal attribution	● Nature, inexplicable, hazardous and its connection with God (and the consequences)	“Life is like that, it’s a thing that is nature” […] “I don’t understand that” […] “That, that, that, no, that, no, that’s what has to happen, happens” UM3 What are they going to do? As long as God is not the only one… UH9 “I don’t know why” UM2 “things of nature” UM1 “bad because God wants” […] “the majority who get bad is because it is written like this” […] “you are bad because God sends it to you” UH3 Passivity in preventive action: “(to be healthy) I do nothing. It’s just like that and that’s it” UM1 “To be healthy? Follow as I go” UM2 “I also had an ulcerative colitis disease, but I believe in my God for my health… and I’m perfect right now. […] God is going to save me, he can do it” LM1 - Understanding the disease: “I don’t know that you have to get sick, but I haven’t stopped to think why, I don’t know.” UM4 “and that’s another thing, because God wills it. I don’t… I can’t tell you anything else” LM1
	● What is not mentioned does not exist	“I don’t know many who take x-rays or analyses, because the less we know the better, the diseases” […] “I don’t know, and I don’t want to know” UH2
	● The temporality - Worrying vs acting - Temporal sequence	- Worrying vs. Dealing “I see them that they are very overwhelmed (to the general people) by many circumstances, of life. What happens? When a person worries, he is preparing his mind that something bad is going to happen to him. It’s the complete opposite of being optimistic. It’s pessimistic. Worrying is synonymous with pessimism and that causes them a lot of stress. […] the gypsies, because we have a greater optimism. There comes a circumstance, well, we give him the solution and that’s it. We deal with the circumstance but we never have in mind that something bad is going to happen to us. “The general people has many worries, not occupations” JH1 “The general people thinks of sickness, of evil, and the Gypsy does not. The gypsy doesn’t even remember that” UH3 “If it’s something that you already feel bad about, that bothers you a lot, the most normal thing is to go to the doctor” UH9 “It’s just that imagining long-term life for a gypsy is complicated.” UH8 -Time sequence “[…] “part of the mouth is destroyed” UM1 “now with the pregnancy I’m being stung a little bit some molars” LM2 “my grandfather knew it was wrong, wrong, wrong, because he did things he had never… done before” UH2
	● Source of information	- Mouth to mouth “Now the glass has broken from here below and they say that it goes up more if you put them on with the broken glass” […] “I have heard say that the children do, because they say that the blood is compatible and the children go out badly, well it can be” UM5 “They say a glass of wine every day says it’s good, I’ve heard that from people” UH2 “It is that I do not know the system of the problem that <…> they say that they are hot, that they do not feel strong <…> it is that they do not:: I do not know it” UH1 - Priest “I’m consulting the pastor of my church and he detects it, says it’s not depression what you have, it’s a little anxiety, it’s fears and fears ”However, there is a polarized discourse with the figure of the shepherd. It conveys a feeling that things depend on God and therefore control does not depend on you and therefore you must not do anything special to get health. “this of now of the gypsies massively turning over to religion and such, is also making the gypsy abandon himself to nature” UH4
